# Transcatheter closure of paravalvular leakage through multiple approaches after surgical mechanical valve replacements: A retrospective study

**DOI:** 10.1097/MD.0000000000040600

**Published:** 2024-11-22

**Authors:** Yang Liu, Chennian Xu, Ping Jin, Mengen Zhai, Hao Tang, Zhiyuan Tian, Anguo Wen, Rui Qiao, Jian Yang

**Affiliations:** a Department of Cardiovascular Surgery, Xijing Hospital, Air Force Medical University, Xi’an, Shaanxi Province, China; b Department of Pharmacology, Key Laboratory of Gastrointestinal Pharmacology of Chinese Materia Medica of the State Administration of Traditional Chinese Medicine, School of Pharmacy, Air Force Medical University, Xi’an, Shaanxi Province, China; c Department of Clinic, The 79th Group Military Hospital of the Chinese People’s Liberation Army, Liaoyang, Liaoning Province, China; d Department of Gastroenterology, Hospital of the Northern Theater Command Air Force, Shenyang, Liaoning Province, China; e Department of 1st Cadre Ward, General Hospital of the Northern Theater Command, Shenyang, Liaoning Province, China.

**Keywords:** 3D printing, paravalvular leak, surgical mechanical valve replacements, transcatheter closure

## Abstract

**Background:**

Transcatheter closure of percutaneous paravalvular leak (PVL) is a technically challenging procedure, especially after surgical mechanical valve replacements (SMVR), as the risk of interference with the prosthetic valve discs and the complex interventional techniques required for mitral PVL closure. Our study was designed to review the results with transcatheter closure of PVL after SMVR.

**Methods:**

From January 2018 through December 2023, a total of 64 patients with PVL after SMVR underwent transcatheter closure with the help of preoperative 3-dimensional printing model and simulator for image evaluation. We reviewed the catheter techniques, perioperative characteristics, and prognosis.

**Results:**

The median follow-up was 28 (3–58) months. The procedure was successful in 60/64 (93.8%) patients. There were 36 aortic PVLs, 27 mitral PVLs, and 1 combined aortic and mitral PVL which were repaired by the transcatheter approach. A total of 24 patients had aortic valve replacements and 20 patients had mitral valve replacements, while 20 patients had previously combined aortic and mitral valve replacements. Procedural time was 35 to 300 (106.6±51.2) minutes. Fluoroscopic time was 8 to 50 (17.6±11.3) minutes. The hospital stay was 5 to 21 (8.1±3.5) days. A total of 47 (78.3%) patients improved by ≥1 New York Heart Association functional class at the 1-year follow-up.

**Conclusion:**

Complex catheter techniques were included in percutaneous closure of mechanical PVL. However, this minimally closure treatment could provide reliable outcomes and shorter hospital stays in selected patients.

## 1. Introduction

PVL has an incidence of 0.5% to 7% in aortic PVL and 5% to 10% in mitral PVL which is a complication after prosthetic valve replacement.^[1–3]^ Recently, transcatheter closure of PVL has emerged as an alternative treatment for patients with high surgical risk.^[4–6]^

In previous studies,^[7–10]^ most transcatheter PVL patients had bioprosthetic valves. However, more than 80% of patients in China had mechanical valves. The construction of mechanical valves is different from that of bioprosthetic valves. The mechanical valves have a shorter frame and closely related leaflets, which greatly increases the risk of interference with occluders.^[11,12]^ Additionally, the techniques for mitral PVL closure after mechanical valve replacement, as interfering with the mechanical aortic valve can affect its function and lead to severe hemodynamic deterioration. In our study, we determined the perioperative outcomes and mid-term follow-up results of transcatheter closure of a PVL associated with surgical mechanical valve replacements (SMVR).

## 2. Materials and methods

### 2.1. Patient population

The study protocol was approved by the ethics committee of Xijing Hospital (Approval Number: KY20150205-1) and registered in the ClinicalTrials.gov Protocol Registration System (NCT02917980). This study is a secondary study^[13]^ while it focused on the transcatheter closure of PVL after SMVR.

**Figure 1.**
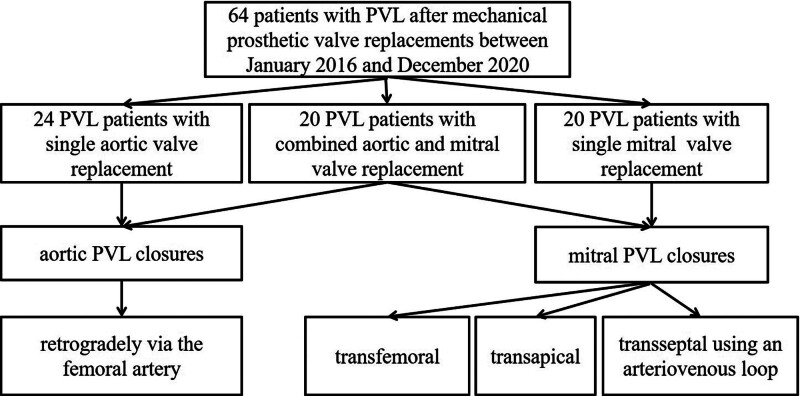
The types of perivalvular leakage and the approach of interventional closure in 64 patients.

Between January 2018 and December 2023, a total of 64 patients with PVL after SMVR underwent transcatheter closure at 5 cardiac centers in China which included Xijing Hospital, Hanzhong Hospital, Anzhen Hospital, Chengdu General Hospital and First Hospital of Zhengzhou University. All the patients provided consent to participate in the study and all clinical documents were reviewed for analysis (Fig. 1).

Twenty-four patients had accepted aortic valve replacements, 20 patients had accepted mitral valve replacements, and 20 patients had accepted combined aortic and mitral valve replacements. All the patients had SMVR. Thirty-six aortic PVLs, 27 mitral PVLs, and 1 combined aortic and mitral PVL were repaired during the transcatheter procedures. Patients were advised of the procedural risks and options and of the off-label use of all closure devices and all signed the informed consent forms. Patient demographics and medical histories are collected (Table 1).

**Table 1:** Preoperative demographic and clinical characteristics.

Variables	Patients (n = 64)
Gender, male	49 (76.6%)
Age, yr	17 to 70 (49.5 ± 10.3)
Previous procedure	
Aortic valve replacement	24 (37.5%)
Mitral valve replacement	20 (31.3%)
Combined aortic and mitral valve replacement	20 (31.3%)
Time since valve replacement, years	0.2 to 14 (4.7 ± 3.4)
History of endocarditis	13 (20.3%)
Hemolysis	38 (59.4%)
NYHA FC II	8 (13.0%)
NYHA FC III	39 (60.9%)
NYHA FC IV	17 (26.1%)
LVEF	
<40	18 (28.6%)
40 to 50	23 (35.9%)
>50	23 (35.9%)
PVL severity	
Mild	0
Moderate	9 (14.1%)
Moderate to severe	31 (48.4%)
Severe	24 (37.5%)
Comorbidities	
Pulmonary hypertension	16 (25%)
Systemic hypertension	8 (12.5%)
Atrial fibrillation	24 (37.5%)
Coronary artery disease	3 (4.7%)
Chronic renal insufficiency, Creatinine > 1.5 mg/dL	7 (10.9%)
EuroSCORE II	
0 to 2	2 (3.1%)
3 to 5	41 (64.1%)
>6	21 (32.8%)

Abbreviations: LVEF *=* left ventricular ejection fraction, NYHA FC *=* New York Heart Association functional class, PVL *=* paravalvular leak.

Note: Categorical variables are presented as frequency (%); continuous variables are presented as mean ± standard deviation when normally distributed. NS: No significant difference. The degree of paravalvular regurgitation was graded semi-quantitatively using Doppler echocardiography and color-flow imaging (mild: <5 mL; moderate: 5 to 8 mL; moderate to severe: 8 to 12 mL; severe: >12 mL). When multiple jets were present, the amounts of regurgitation from the separate jets were totaled for semi-quantitation.

### 2.2. Procedure

All the procedures were performed in the biplane catheterization laboratory. Before the procedures, the location of the PVLs and the volume of the regurgitation were confirmed by 3-dimensional transesophageal echocardiography (TEE), transthoracic echocardiography (TTE) or CT angiography in selected patients. A total of 6 patients with mitral PVL closures were performed via the transapical approach under general anesthesia. All the other procedures were performed under local anesthesia. The approaches retrogradely via the femoral artery were used in all the aortic PVL closures. The multiple approaches were used in mitral PVL closures, including transfemoral, transapical, and transseptal using an arteriovenous loop.

**Figure 2.**
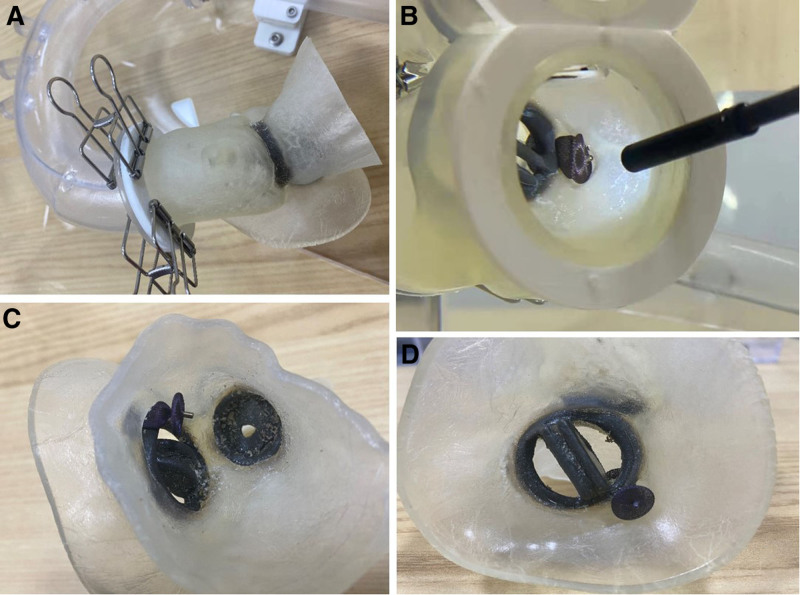
Preoperative 3D printing model and simulator were used to simulate the interventional occlusion process. (A) The whole aortic root model was established by 3D printing technology. (B–D) After placing the occluder at the perivalvular leakage position in vitro, the position of the occluder and its relationship with the mechanical valve from different angles was observed.

According to the preoperative CT results, the heart team can reconstruct the 3D printed models of the anatomical structure of PVLs and simulate PVL intervention in vitro simulator, thus the operator use the 3D printing preprocedural information to plan for a safe PVL occluding procedure (Fig. 2).

### 2.3. Transcatheter closure of aortic mechanical PVL

**Figure 3.**
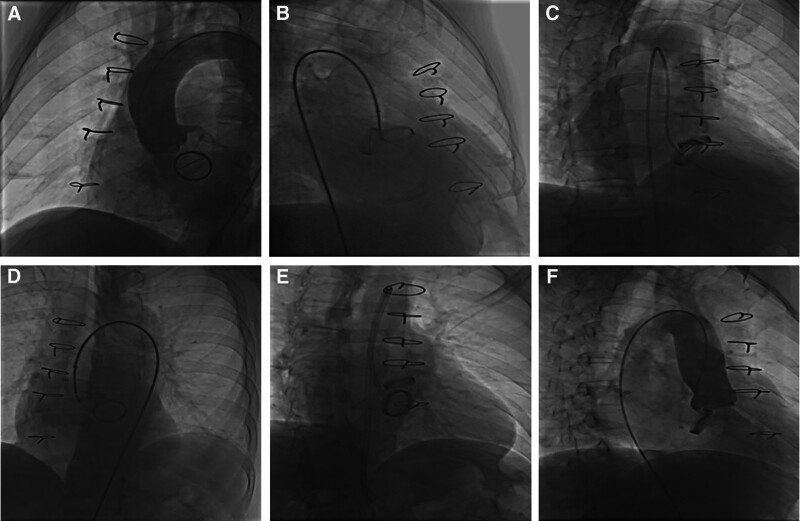
Angiography profiling transcatheter closure of an aortic mechanical PVL. (A) Ascending aorta angiogram to profile the para-aortic regurgitation. (B) Retrograde crossing of the PVL with a guidewire. (C) Retrograde crossing of the PVL with a guidewire. (D) Adjust the perspective angle to confirm that the guide wire passes through PVL. (E) Occluder device placed at the position of the PVL. (F) Ascending aorta angiogram after deployment. PVL = percutaneous paravalvular leak.

The procedures were performed retrogradely via the femoral artery in all patients with aortic PVL. A 6 French (Fr) arterial access, a 5 Fr multipurpose diagnostic catheter (Boston Scientific Corporation, Marlborough) and a 260-cm (0.032-in) straight-tip wire (Terumo Corporation, Tokyo, Japan) were advanced through the PVL after an initial aorta angiographic scan confirmed aortic regurgitation and the location of the PVLs. Then it was placed with an extra stiff, 0.035-inch exchange-length Lunderquist guidewire (Cook Medical, Bjaeverskov, Denmark) through the defect into the left ventricle, followed by the placement of a relatively larger transducing sheath (Cook Medical, Bjaeverskov, Denmark) over the guidewire, through which the appropriate Amplatzer occluder device (AGA Medical Corp., Plymouth) was deployed. According to the size and shape of defect, multiple devices were choosen (Fig. 3).

### 2.4. Transcatheter closure of mitral mechanical PVL

The best choice for patients with pure SMVR was the retrograde approach via the femoral artery. The para-mitral regurgitation and the location of defect were confirmed by the left ventricular angiogram, while a 6 Fr pigtail catheter was placed in the left ventricle via femoral arterial access. Then a 5 Fr multipurpose diagnostic catheter and a 260-cm (0.032-in) Terumo straight-tip wire were advanced through the defect based according to the results of the angiogram. An extra stiff, 0.035-in exchange-length Lunderquist guidewire (Cook Medical, Bjaeverskov, Denmark) was placed through the aortic valve and the para-mitral defect into the left atrium, followed by the placement of a relatively larger transducing sheath over the guidewire. Then the appropriate Amplatzer occluder device was deployed.

**Figure 4.**
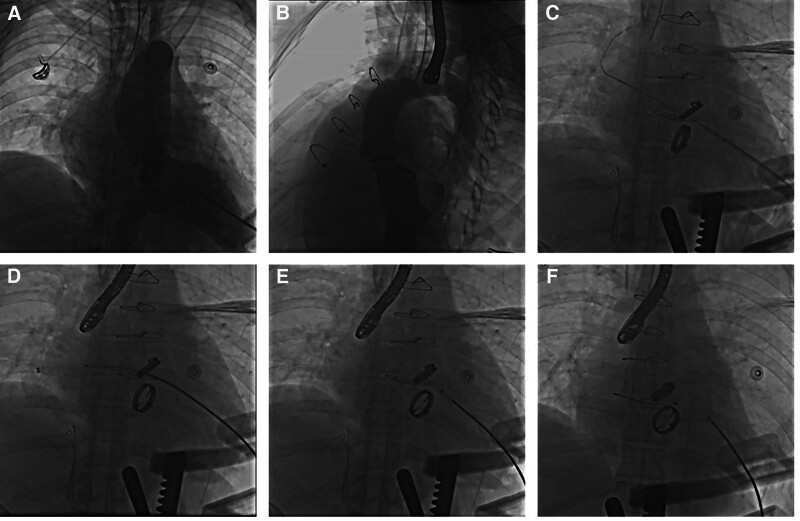
Angiography during the transcatheter procedure of mitral mechanical PVL closure via multiple approaches. Mini-invasive transapical approach. (A) The transapical access was obtained with a 6 Fr sheath. Left ventricular angiogram to profile the para-mitral regurgitation. (B) Left ventricular angiogram to profile the para-mitral regurgitation. (C) Guide wire through PVL. (D) Guide wire through PVL. Occluder placed at the position of the PVL. (E) The mitral PVL crossed retrogradely with the guidewire. (F) The occluder was deployed. PVL = percutaneous paravalvular leak.

For 12 patients, advancing the delivery sheath or catheter through the defect was difficult from the retrograde femoral approach. Then a transseptal puncture was performed, and an arteriovenous loop was established by snaring the wire in the left atrium and externalizing it through the femoral vein. The delivery sheath was advanced, and devices were deployed (Fig. 4).

For 4 patients who underwent combined mitral and aortic valve replacement, closure of the mitral PVL could not be performed retrogradely via the femoral artery as they had the mechanical prosthetic valve as a goalkeeper. Therefore, the para-mitral defect was approached retrogradely via the transapical access. A left minithoracotomy was performed with apical cardiac exposure in the patient under general anesthesia. After placing the pledgets, the transapical access was obtained with a 6 Fr sheath placed in the standard fashion at the apex. Through the 6 Fr sheath, a 5 Fr multipurpose diagnostic catheter and a 260-cm (0.032-in) Terumo straight-tip wire were passed through the mitral PVL under fluoroscopic guidance after the left ventricular angiogram confirmed aortic regurgitation and the location of the defect. Then, the 6 Fr short sheath was exchanged for a relatively larger sheath, and the appropriate Amplatzer occluder device was deployed. After the device was released, we took a left ventricular angiogram. Multiple devices might be used if over-moderate paravalvular regurgitation was noticed. The percutaneous transapical approach was performed for the other 2 patients with mitral PVL after combined mitral and aortic valve replacement without a thoracotomy.

For the resean that devices designed for percutaneous closure of PVL are not available except for Occlutech, all Amplatzer occluder devices used in this study were used off-label for PVL closure, such as the Amplatzer atrial septal occluder, the Amplatzer muscular ventricular septal defect occluder, the Amplatzer duct occluder, and the Amplatzer vascular plugs (AVP II) (AGA Medical Corp., Plymouth), Amplatzer Valvular Plug III (Abbott Medical Corp., Chicago).

### 2.5. Perioperative outcome and follow-up

All clinical data were reviewed, and perioperative characteristics were documented, such procedural time, fluoroscopic time, and postoperative hospital stay, among others. All patients were seen in the clinic to ascertain their clinical status (New York Heart Association functional class [NYHA]) and adverse events after discharge. At 3 months, 6 months and 12 months after the procedures, the transthoracic echocardiographs were done. The CT angiography was also used during the follow-up period.

### 2.6. Statistical analysis

All the statistical analyze were conducted with SPSS 22.0 software (IBM SPSS Statistics for Macintosh, Version 22.0, IBM Corp, Armonk). Continuous variables are presented as means ± SD while categorical variables are expressed as percentages. Univariable comparisons were performed with the Student unpaired *t*-test for continuous normally distributed data and the chi-square test was used for categorical data. Values of *P* < .05 were considered with statistical significance.

## 3. Results

### 3.1. Procedural and in-hospital outcomes

Procedural success was achieved in 60/64 (93.8%) patients who underwent percutaneous closure of mechanical PVLs. Thirty-six aortic PVLs, 27 mitral PVLs, and 1 combined aortic and mitral PVL were repaired during the transcatheter procedures. Multiple devices were used such as patent ductus arteriosus occluders, muscular ventricular septal defect occluders, and AVP II occluders. In 1 patient with a mitral PVL, the sheath could not be advanced across the defect. In 1 patient with an aortic PVL and 2 others with mitral PVLs, the maximum 20 mm AVP II or ADO occluders were deployed at the defects. However, the occluders were not stable and could be easily pulled back into the aorta or the left atrium during a push–pull test, then the procedures were terminated and changed to open surgery later. No hospital deaths occured. The procedural characteristics are collected (Table 2).

**Table 2:** The procedural characteristics.

Total patients	64
Patients	
Acute successful procedures	60 (93.8%)
Aortic PVL	36
Mitral PVL	27
Combined aortic and mitral PVL	1
Approach	
Aortic PVL	
Transfemoral	36
Mitral PVL	
Transfemoral	10
Trans-septal A-V loop	12
Transapical	4
Transseptal and Transapical A-V loop	2
Patients with different devices	
PDA occluder	16
ADO II	2
VSD occluder	6
AVP II occluder	40
Single device	50
Two devices	13
Three devices	1
General anesthesia	6
Local anesthesia	58
Fluoroscope time (min)	8 to 50 (17.6 ± 11.3)
Procedural time (min)	35 to 300 (106.6 ± 51.2)
Hospital stay (d)	5 ± 21 (8.1 ± 3.5)
Patients needing blood transfusion	7 (10.9%)

Abbreviations: ADO = amplatzer duct occluder, A-V = arteriovenous, AVP = amplatzer vascular plug, PDA = patent ductus arteriosus, PVL = percutaneous paravalvular leak, VSD = ventricular septal defect.

PVL regurgitation decreased to mild or moderate-mild immediately after the procedure in all successfully treated patients. Four patients had hemolysis after the procedure. Among the 4, 2 patients had acute renal insufficiency and needed continuous renal replacement therapy and blood transfusions. All these patients recovered before discharge. Other complications included 2 femoral pseudoaneurysms and 1 hemothorax after the transapical approach. All of these patients recovered before discharge.

### 3.2. Follow-up

**Figure 5.**
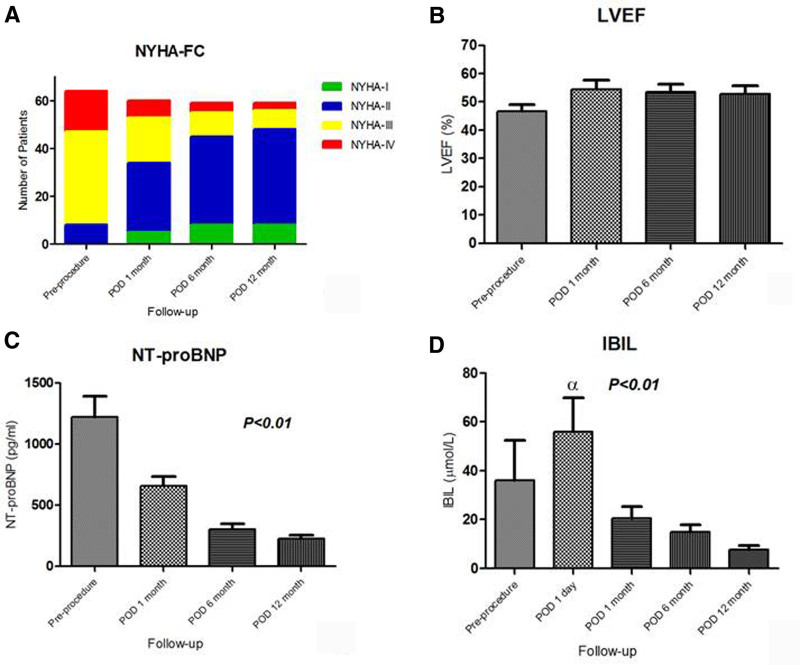
Clinical and laboratory examination results before and after the transcatheter procedures. (A) Improvement in New York Heart Association functional class during the 1-year follow-up period. (B) The left ventricular ejection fractions during the 1-year follow-up period. (C) NT-proBNP levels during the 1-year follow-up period. (D) The indirect bilirubin levels during the 1-year follow-up period.

**Figure 6.**
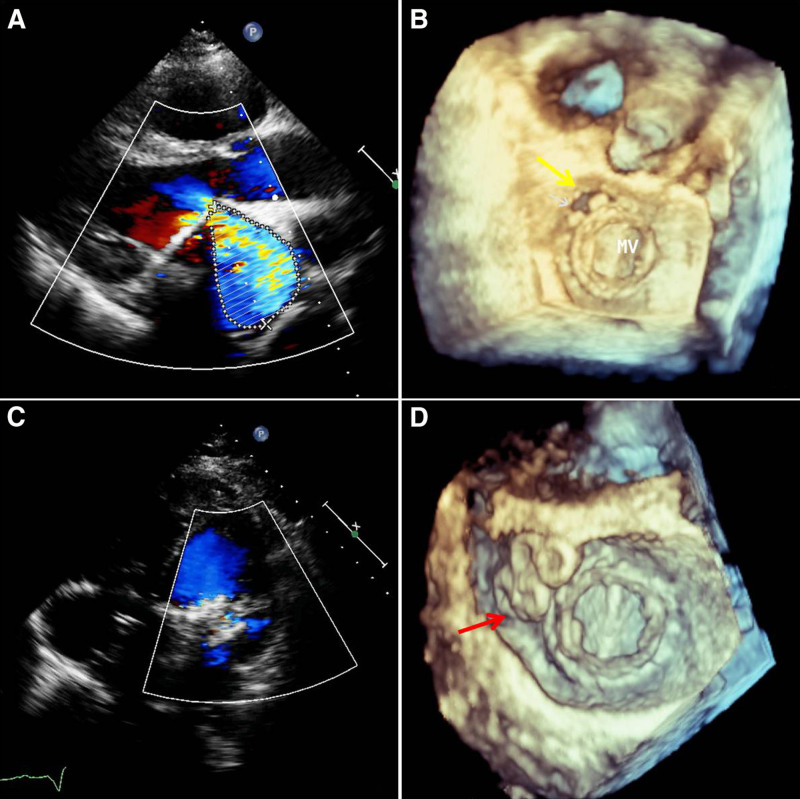
Echocardiography before the procedure and during the follow-up period. (A) 2-dimensional transesophageal echocardiography shows the mitral PVL before the procedure; the color jet shows the PVL regurgitation. (B) 3-dimensional transesophageal echocardiography shows the mitral PVL before the procedure. (C) 2-dimensional transesophageal echocardiography shows the occluder and no residual regurgitation during the follow-up period. (D) 3-dimensional transesophageal echocardiography shows the mitral PVL closed with the occluder (the *yellow arrow* shows the PVL. The *red arrow* shows the occluder). PVL = percutaneous paravalvular leak.

**Figure 7.**
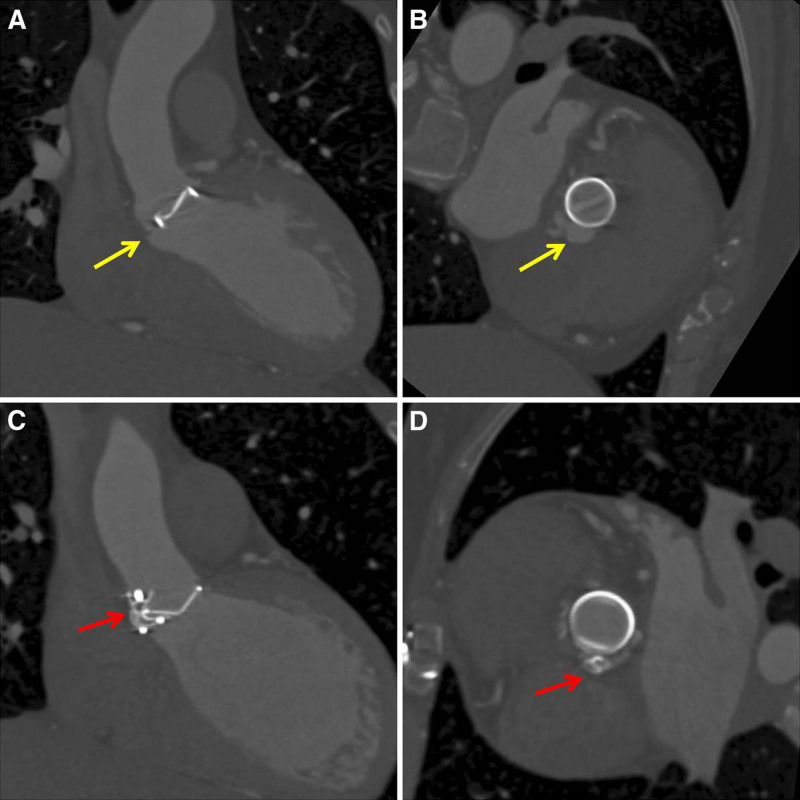
Computed tomography angiography before the procedure and during the follow-up period. (A) The aortic PVL before the procedure, sagittal view. (B) The aortic PVL before the procedure, axial view. (C) The aortic PVL was closed using the occlude, sagittal view. (D) The aortic PVL was closed with the occlude, axial view (the *yellow arrow* points to the PVL. The *red arrow* indicates the occluder). PVL = percutaneous paravalvular leak.

The median follow-up period was 28 (3–58) months, and follow-up was 100% complete. Forty-seven (78.3%) patients improved by ≥ 1 NYHA functional class at the 1-year follow-up visit. The left ventricular ejection fractions showed no significant improvement. However, the NT-proBNP levels decreased to normal in most patients, and the indirect bilirubin level decreased significantly after the procedure (Fig. 5). Most patients had nothing more than mild to moderate paravalvular regurgitation as seen on TEE, TTE, or CT angiography follow-up scans (Figs. 6 and 7). Three patients had recurrent hemoglobinuria in the first 2 months after discharge. Among them, 1 patient had severe anemia. Valve re-replacement was performed for this patient 2 months after discharge. The occluder interfered with the disc of the mechanical valve. This patient died of low cardiac output syndrome after open-heart surgery. The other 2 patients recovered uneventfully in 2 months.

## 4. Discussion

Paravalvular regurgitation is a common complication after SMVR. Redo open-heart surgery to repair the PVL or for valve re-replacement has traditionally been the gold standard^[14]^ while it is a big challenge. Compared with surgical procedures, percutaneous transcatheter methods have several advantages. Re-sternotomy and cardiopulmonary bypass are avoided, which could contribute to decreasing the incidence of the main complications after redo open-heart surgery.^[15,16]^ General anesthesia was avoided as well as times of the procedure and hospital stays after the procedures were greatly shortened.^[17]^

**Figure 8.**
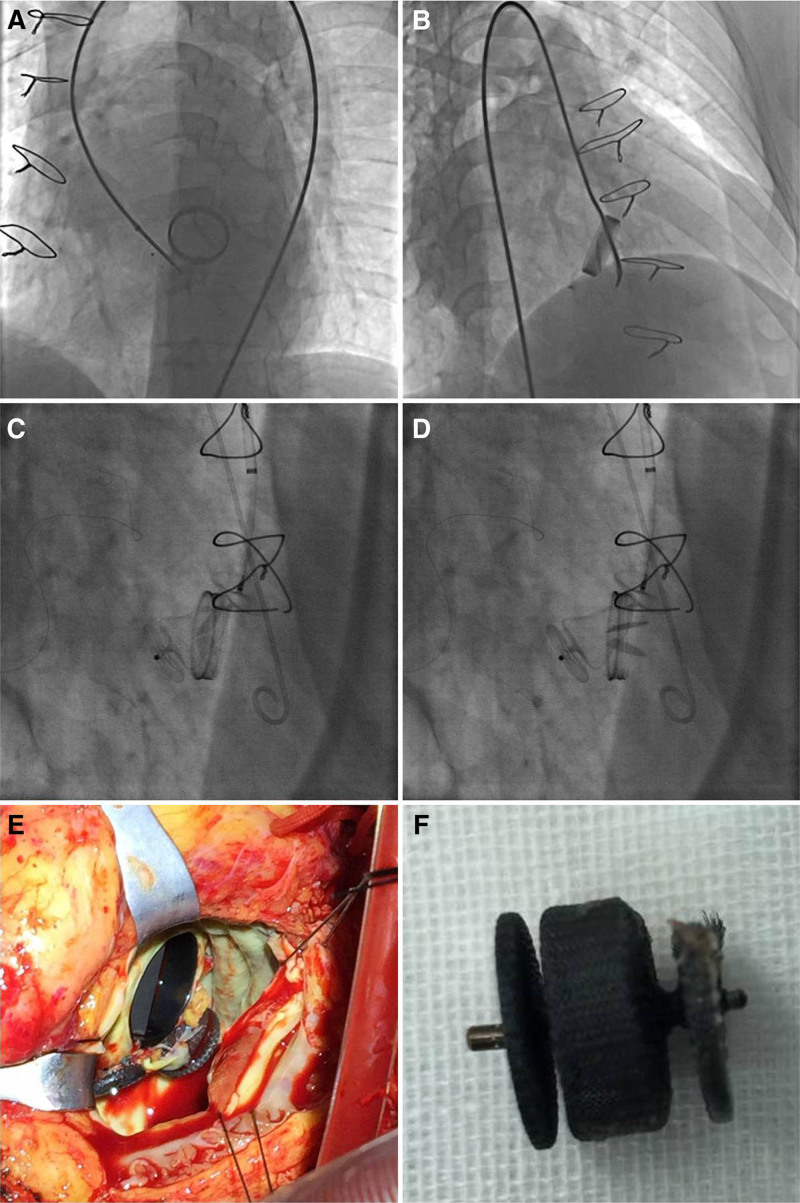
The interference of the occluder with the mechanical valve disc. (A) The mechanical mitral valve could not close because the occluder interfered with the mechanical valve disc. (B) The mechanical mitral valve could not open because the occluder interfered with the valvular disc. (C) The mechanical mitral valve closed normally with no interference. (D) The mechanical mitral valve could open normally because there was no interference. (E) The position of the occluder had changed and interfered with the valvular disc during the redo open operation after transcatheter closure. (F) The worn occluder was removed.

Currently, reoperation is no longer the first choice in our institute, as the in-hospital mortality rate is higher than that with interventional PVL closure.^[13]^ However, transcatheter PVL closure need an experienced heart team with abundant catheter, especially for SMVR patients. Meanwhile, the risk of PVL increases with the use of mechanical heart valves compared with the use of bioprosthetic valves.^[11,12]^ Furthermore, the construction between mechanical valves and bioprosthetic valves is different. Therefore, the open-and-close status of the mechanical valve disc should be carefully monitored immediately after the occluder is deployed. Multiple projective positions might be needed to investigate whether the valvular disc can open-and close normally without any interference from the occluder. Figure 8A-D shows the different situations after insertion of the occluder. The occluder should be repositioned immediately if the valvular disc does not open or close normally. The occluder should be removed or exchanged for other types or sizes if the interference persists. As a result, the interventionist must focus on more technical details for patients with mechanical valves than for those with bioprosthetic valves during the transcatheter procedures, including the sizing of the PVL, the choice of occluder, and the position of the occluder after delivery.

Meanwhile, the migration/displacement of the occluder is another important issue after delivery. Different from the bioprosthetic valve, the mechanical valve has a shorter frame. The devices used for PVL closure usually were higher than the valvular frame. Therefore, the occluder might change its position after delivery with the heart beating. In this study, 1 patient had severe anemia and recurrent hemoglobinuria 2 months later after discharge even though he recovered well after the procedure. Valve re-replacement was performed for this patient. The fact that the device was displaced was discovered during the operation. The axis of the device was angled with the prosthetic valve, and the distal part of the AVP II device was found to interfere with the valvular disc (Fig. 8E). Then the device was maintained until it was removed during the operation (Fig. 8F). Demonstration of the position of the device by TTE, TEE, or CT angiography is mandatory during follow-up visits.

The third technical issue relates to the mechanical mitral valve is the intricate technics required. Different from an aortic PVL, which needs less complicated techniques, percutaneous closure of a mitral PVL can be technically intricate. In addition, transcatheter closure in mitral PVL patients with previous SMVR can be extremely challenging, as passing through the mechanical aortic valve can affect its function and thereby lead to severe hemodynamic deterioration.^[18–20]^ In this study, more patients had aortic PVLs than mitral PVLs. One can access mitral PVLs antegradely and transseptally, transapically, or retrogradely from the femoral artery. It eventually allows the surgeon to pass the sheath through the shallow angles and calcified lesions. According to previous reports,^[4,5,21]^ these procedures are most often done with the patient under general anesthesia and with the guidance of TEE. In this study, all except 6 transapical cases were performed with the patient under local anesthesia under TEE guidance. Based on our experiences, local anesthesia and TTE guidance contribute to successful treatment, allowing most patients to avoid more invasive procedures and hospitals to save medical expenses. In most patients with mitral PVL, a retrograde approach was the first choice. In those after combined MVR and AVR, mitral PVLs were closed via transapical approach, a viable alternative like a transseptal access with steerable sheath provides excellent control and stable support with no need for arteriovenous looping. Steerable sheaths are safe and effective devices that support mitral PVL closure, particularly in cases with challenging PVL locations.^[22]^

Last, residual regurgitation can limit the therapeutic effect because of hemolysis and congestive heart failure that remain after PVL closure. The incidence of residual regurgitation was significantly lower in patients who had surgery than in those who had a transcatheter procedure.^[6,8,22–24]^ In this study, mild to moderate residual regurgitation appeared in the first several cases as the surgeons were in their learning curve and not familiar enough with the PVL anatomy and which devices to use. However, more than mild residual regurgitation and remaining hemolysis were seldom found thereafter. The choice of device was based on the results of preprocedural TEE or TTE scans and angiography scans taken during the procedures. The large crescent-shaped leak was closed either with 1 large device (e.g., a PDA occluder or an ADO II) or multiple AGA PLUG II devices. For the long tunnel-shaped leak with a large central cavity, we prefer to use a single AGA PLUG II device. The recent review from Fabian Nietlispach gave physicians specific suggestions on the choice of devices.^[15,25,26]^

## 5. Limitations

The present case series is a retrospective, nonrandomized study in selected centers with its inherent limitations. In any case, further studies are necessary to evaluate the long-term results.

## 6. Conclusions

Based on this multiple-center experience, transcatheter closure of mechanical paravalvular leaks can provide a safe, minimally invasive treatment with reliable in-hospital outcomes and shorter hospital stays in selected patients.

## Author contributions

**Conceptualization:** Yang Liu, Chennian Xu.

**Data curation:** Yang Liu, Chennian Xu, Ping Jin.

**Formal analysis:** Yang Liu, Chennian Xu, Ping Jin.

**Funding acquisition:** Yang Liu, Chennian Xu, Jian Yang.

**Investigation:** Chennian Xu.

**Project administration:** Jian Yang.

**Supervision:** Hao Tang, Rui Qiao, Jian Yang.

**Validation:** Mengen Zhai, Hao Tang, Zhiyuan Tian, Anguo Wen, Rui Qiao.

**Writing – original draft:** Chennian Xu, Yang Liu.

**Writing – review & editing:** Chennian Xu, Jian Yang.
